# Association between weekday sleep, weekday-weekend sleep duration gap, and hypertension in American adolescents: a nationwide cross-sectional study

**DOI:** 10.3389/fpsyt.2024.1470121

**Published:** 2024-12-23

**Authors:** Yan Luo, Qingyuan Li, Tong Feng, Ran Duan, Yingyi Chen

**Affiliations:** ^1^ Respiratory Department, Chengdu Xindu District Second People's Hospital, Chengdu, China; ^2^ School of Clinical Medicine, Chengdu Medical College, Chengdu, Sichuan, China; ^3^ Respiratory and Critical Care Department, The First Affiliated Hospital of Chengdu Medical College, Chengdu, Sichuan, China; ^4^ The Second School of Clinical Medicine, Southern Medical University, Guangzhou, China; ^5^ Onology Department, The First Affiliated Hospital of Chengdu Medical College, Chengdu, Sichuan, China; ^6^ Dermatological Department, The First Affiliated Hospital of Chengdu Medical College, Chengdu, Sichuan, China

**Keywords:** weekday-weekend sleep duration gap, hypertension, sleep patterns, cross-sectional study, NHANES

## Abstract

**Background:**

The increasing prevalence of primary hypertension among children and adolescents is a global health concern, with inadequate sleep duration identified as a significant risk factor. This study investigates the impact of weekday-weekend sleep duration gap (WWSDG) on hypertension among American adolescents.

**Methods:**

Using data from the NHANES 2017-2020 cohort, we analyzed sleep patterns and hypertension prevalence among 430 adolescents. Sleep durations on weekdays and weekends were used to calculate WWSDG. Hypertension was defined according to the American Academy of Pediatrics guidelines.

**Results:**

The study found that among 430 American adolescents, shorter weekday sleep duration (less than 8 hours) was significantly associated with an increased risk of hypertension (OR: 1.092, 95% CI: 1.042–1.144), while extended weekend sleep did not show a protective effect. Additionally, longer total weekly sleep duration (over 10 hours) was linked to a reduced risk of hypertension, but the difference in sleep duration between weekdays and weekends was not significantly correlated with hypertension risk.

**Conclusions:**

The study highlighted that insufficient weekday sleep duration significantly increases hypertension risk among adolescents. Contrary to popular belief, compensating for sleep deprivation through extended WWSDG did not mitigate this risk. Public health interventions should focus on promoting consistent and adequate sleep throughout the week for cardiovascular health benefits in adolescents. Further longitudinal research is necessary to explore causal relationships and the long-term effects of sleep variability on health outcomes.

## 1 Introduction

The increasing prevalence of primary hypertension in children and adolescents is a significant global health issue. While approximately four percent of six-year-olds are affected, this rate rises substantially among obese adolescents, with nearly one in six experiencing hypertension (1). Among the various risk factors associated with adolescent hypertension, sleep problems, particularly inadequate sleep duration, have been consistently linked to its development (2, 3). It is generally recommended that adolescents aged 13-18 get 8-10 hours of sleep per day (4); however, the average sleep duration for American teenagers ranges from 7.5 to 8.5 hours daily (5), with many getting less than 7 hours on school nights (6).

Hypertension in adolescents is an increasingly prevalent public health issue with substantial implications for future cardiovascular health. Insufficient sleep has been identified as a key modifiable factor associated with elevated blood pressure, particularly among youth. Adolescents frequently experience limited sleep on weekdays due to early school start times and social obligations, often attempting to compensate by sleeping longer on weekends. This pattern, known as the weekday-weekend sleep duration gap (WWSDG), reflects the difference between average sleep duration on weekdays and weekends. A survey of middle-aged adults in Korea revealed that catching up on sleep over the weekend was associated with a reduced risk of hypertension (7).

Recent research, however, challenges the assumption that extended weekend sleep can effectively counterbalance the adverse effects of insufficient weekday sleep. Studies by Putilov and colleagues have demonstrated that weekend sleep extension may not significantly restore health markers compromised by weekday sleep loss. Furthermore, analyses separating weekday sleep duration from the WWSDG suggest that weekday sleep itself, rather than the variability between weekday and weekend sleep, is a primary contributor to health outcomes (8, 9). 

In light of these findings, this study investigates the independent associations of weekday sleep duration and WWSDG with hypertension in a representative sample of American adolescents. By analyzing these sleep variables separately, we aim to clarify whether WWSDG has a distinct relationship with hypertension risk.

## 2 Materials and methods

### 2.1 Study population

**Figure 1:**
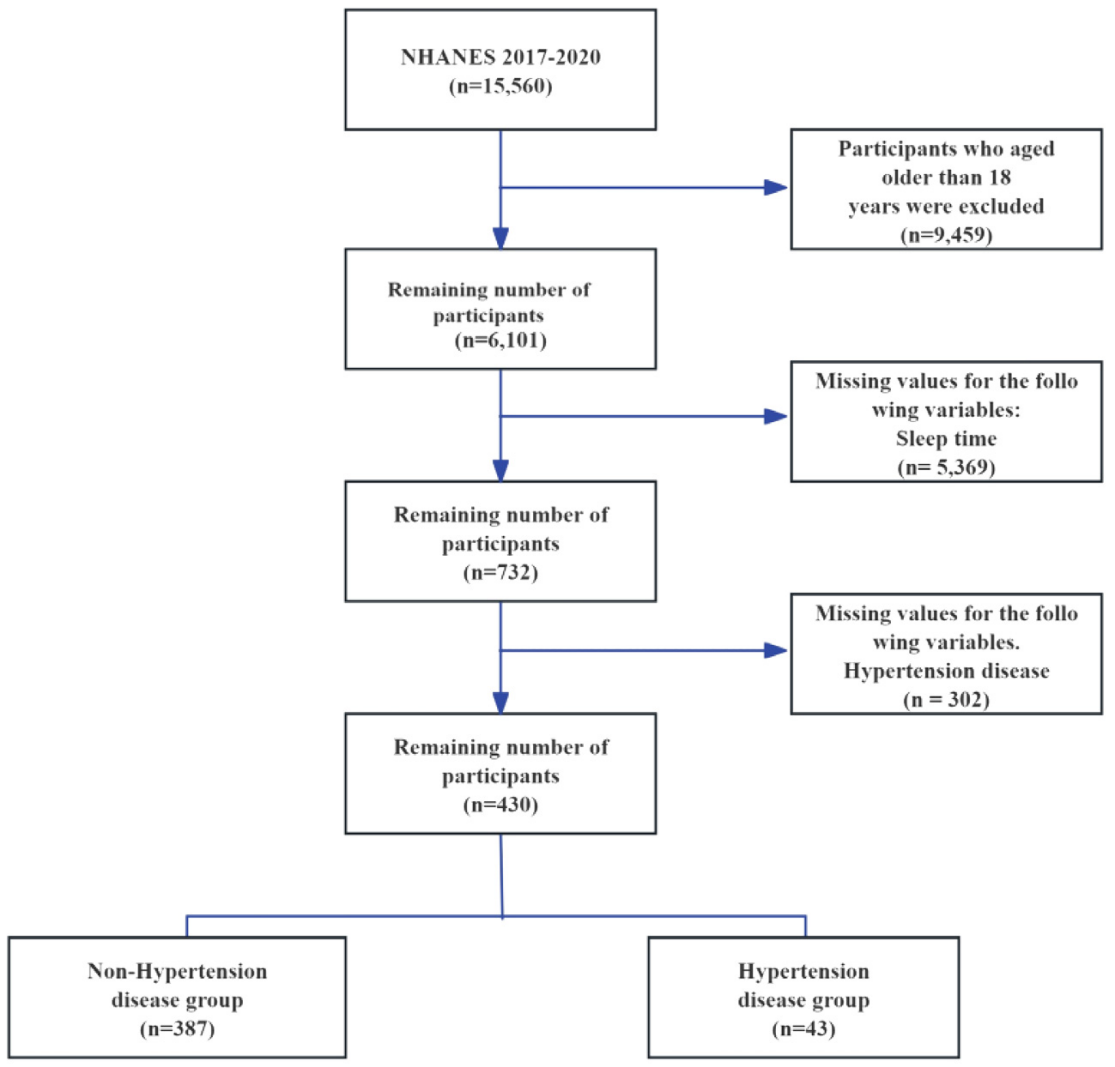
Flow chart for participant selection for the analysis.

This study leverages the publicly accessible NHANES database, which evaluates the health and nutritional status of American adults and children through a comprehensive survey that addresses a variety of populations and health topics. The NHANES database encompasses demographic, dietary, physical examination, laboratory, questionnaire, and restricted access data. Given that previous surveys omitted weekend sleep duration, this research includes data spanning from 2017 to 2020. The study sample comprises individuals who completed the sleep questionnaire. Initially, the NHANES 2017-2020 cohort contained 15,560 participants. Exclusions were made for those exceed 18 years old (9,459 participants), those missing sleep duration data (5,369 participants), and those with no recorded hypertension events (302 participants), resulting in a final sample of 430 individuals (**Figure 1**). Of these, 43 were diagnosed with hypertension, while 387 were identified as normotensive. This study was written in accordance with the STROBE checklist, detailed in **Supplementary Table S1**.

For more detailed information about the survey design, methodology, and data used in this study, interested individuals can visit the NHANES website at https://www.cdc.gov/nchs/nhanes/. It is important to note that the NHANES procedure has received authorization from the National Center for Health Statistics, and all participants involved in the survey have provided written consent.

### 2.2 Assessments of WWSDG

In the NHANES 2017-2020 survey, the WWSDG was quantified based on the reported sleep durations of participants on weekdays (Monday through Friday) and weekends (Saturday and Sunday). The questions posed to determine sleep patterns were as follows: During weekdays, participants generally go to bed and wake up at what specific times? How about on weekends – what are the typical sleep and wake-up times for participants during those days? The overall amount of sleep over the course of the week was determined by taking into account the different durations of sleep on weekdays and weekends. This was calculated using the formula: (5 times the amount of sleep on weekdays plus 2 times the amount of sleep on weekends) divided by 7. WWSDG was calculated by subtracting the average weekday sleep duration from the weekend sleep duration. A normal WWSDG duration was defined as a difference of 0 hour or less between weekend and weekday sleep durations. If the WWSDG duration exceeds 0 hour, with weekend sleep exceeding weekday sleep by more than 0 hour, it is classified as significant WWSDG. The specific coding and questions related to sleep issues can be found in **Supplementary Table 2**.

### 2.3 Hypertension definition

According to the American Academy of Pediatrics guidelines (10), hypertension is defined as an average clinic-measured systolic blood pressure (SBP) and/or diastolic blood pressure (DBP) at or above the 95th percentile based on age, sex, and height percentiles. Hypertension is further classified into stage 1 and stage 2. For children aged 1 to under 13 years, stage 1 hypertension is defined as SBP and/or DBP from the 95th percentile to less than the 95th percentile plus 12 mmHg, or 130/80 to 139/89 mmHg (whichever is lower). Stage 2 hypertension is defined as SBP and/or DBP at or above the 95th percentile plus 12 mmHg, or 140/90 mmHg or higher (whichever is lower). For children aged 13 years and older, stage 1 hypertension is defined as SBP and/or DBP between 130/80 and 139/89 mmHg. Stage 2 hypertension is defined as SBP and/or DBP at or above 140/90 mmHg.

### 2.4 Covariates

A standardized interview questionnaire was utilized to gather demographic and lifestyle data, including information on age, gender, race/ethnicity (classified as Mexican American/other Hispanic, Non-Hispanic White, Non-Hispanic Black, and Other Race), family income, smoking habits, and alcohol consumption. Economic status was deduced from family income and poverty levels, with higher income and lower poverty indicating a better economic status. Body mass index (BMI) was determined by dividing weight in kilograms by the square of height in meters.

### 2.5 Statistical analyses

Participants were then categorized into groups based on hypertension status for descriptive statistical analysis of standard demographic indicators. Continuous variables, whether normally distributed or not, were represented as mean (standard error) and median (interquartile range) respectively. Multiple group comparisons of all continuous variables in this study were conducted using a weighted linear regression model. Categorical variables were presented as frequencies (percentages) and analyzed using the weighted chi-square tests.

A weighted multifactorial logistic regression analysis was performed to investigate the relationship between WWSDG and hypertension. Model 1 served as a univariate model, while Model 2 additionally adjusted for educational level, income-to-poverty ratio, and BMI.

The statistical analyses were conducted using R software (version 4.1.2, The R Foundation) and EmpowerStats (version 4.1, X&Y Solutions Inc.), with significance considered at a two-sided P-value below 0.05.

## 3 Results

### 3.1 Characteristics of study participants

**Table 1** provides the characteristics of adolescents with and without hypertension. The sample is divided into two groups: non-hypertension (N = 387) and hypertension (N = 43). The study population had an average age of approximately 16.6 years. In terms of gender distribution, males constituted about 50.2% of the non-hypertension group and 46.7% of the hypertension group, while females made up 49.8% of the non-hypertension group and 53.3% of the hypertension group. The prevalence of hypertension in the study population was approximately 10%, with 43 out of the 430 adolescents diagnosed with hypertension. BMI shows significant differences between the groups, whereas other variables such as age, sex, race, and sleep duration do not show significant differences.

**Table 1:** Characteristics of adolescents with and without hypertension.

Characteristic	Non- Hypertension (N = 387)	Hypertension (N = 43)	P value
Age (years)	16.6 (15.3,17.8)	16.8 (15.1,18.2)	0.7054
Sex (%)			0.7503
Male	50.2 (43.4,57.0)	46.7 (24.9,69.9)	
Female	49.8 (43.0,56.6)	53.3 (30.1,75.1)	
Race (%)			0.0764
Mexican American	15.7 (11.1,21.7)	18.1 (4.4,51.9)	
Non-Hispanic White	11.9 (8.0,17.4)	23.3 (11.7,41.0)	
Non-Hispanic Black	52.8 (42.6,62.8)	52.9 (28.1,76.4)	
Other Race	19.6 (14.3,26.3)	5.6 (1.5,18.5)	
BMI (kg/m^2^)	24.6 (23.9,25.3)	29.5 (26.8,32.2)	0.0016
Income to poverty ratio (%)			0.5109
≤1	13.7 (9.8,18.9)	22.8 (6.8,54.3)	
1-3	40.1 (32.8,47.8)	41.7 (19.4,68.1)	
>3	46.2 (38.0,54.7)	35.5 (13.6,65.8)	
Weekday sleep duration (%)			0.3988
≤8h	42.2 (33.6,51.4)	49.2 (26.3,72.5)	
8-10h	51.3 (43.5,59.1)	49.9 (27.3,72.5)	
>10h	6.4 (4.2,9.7)	0.9 (0.1,8.9)	
Weeknd sleep duration (%)			0.5253
≤8h	13.1 (8.8,19.0)	11.1 (3.0,33.3)	
8-10h	58.2 (48.2,67.6)	69.3 (42.1,87.5)	
>10h	28.7 (22.3,36.1)	19.6 (9.1,37.2)	
WWSDG (%)			0.0919
≤0h	25.3 (20.3,30.9)	11.7 (3.8,30.8)	
>0h	74.7 (69.1,79.7)	88.3 (69.2,96.2)	

Median and interquartile range for continuous variables; % for categorical variables.

WWSDG, weekend catch-up sleep; BMI, body mass index.

### 3.2 Associations between WWSDG and hypertension

The association between weekday sleep duration and hypertension reveals a higher risk for individuals sleeping less than 8 hours, with an odds ratio of 1.092 (95% CI: 1.042–1.144) (**Table 2**). However, weekend sleep duration shows no significant association with hypertension (**Table 3**). When considering the whole week, longer sleep durations (more than 10 hours) exhibit a decreased risk of hypertension compared to the 8–10 hours reference group, with an odds ratio of 0.199 (95% CI: 0.049–0.807) (**Table 4**).

**Table 2:** Associations between weekdays sleep duration and hypertension.

	Model 1 OR (95% CI)	Model 2 OR (95% CI)
sleep duration (h)	0.771 (0.545,1.090)	0.749 (0.570,0.984)
Duration of sleep (h)		
8-10h	Reference	Reference
<8h	0.985 (0.721,1.670)	1.092 (1.042,1.144)
≥10h	0.116 (0.012,1.160)	0.207(0.029,1.457)

Model 1: no covariates were adjusted.

Model 2: age, sex, race, income to poverty ratio and BMI were adjusted.

**Table 3:** Associations between weekends sleep duration and hypertension.

	Model 1 OR (95% CI)	Model 2 OR (95% CI)
sleep duration (h)	1.050 (0.890, 1.230)	1.065 (0.895, 1.240)
Duration of sleep (h)		
8-10h	Reference	Reference
<8h	1.480 (0.320, 6.200)	1.860 (0.420, 7.900)
≥10h	0.830 (0.310, 2.080)	0.910 (0.320, 2.590)

Model 1: no covariates were adjusted.

Model 2: age, sex, race, income to poverty ratio and BMI were adjusted.

**Table 4:** Associations between whole week sleep duration and hypertension.

	Model 1 OR (95% CI)	Model 2 OR (95% CI)
sleep duration (h)	0.810 (0.570, 1.120)	0.785 (0.575, 1.015)
Duration of sleep (h)		
8-10h	Reference	Reference
<8h	0.905 (0.310, 2.650)	0.920 (0.330, 2.560)
≥10h	0.300 (0.090, 1.010)	0.220 (0.060, 0.890)

Model 1: no covariates were adjusted.

Model 2: age, sex, race, income to poverty ratio and BMI were adjusted.

**Table 5** shows that WWSDG has a potential association with hypertension, although the results are not statistically significant. In the analysis of continuous WWSDG duration, neither the unadjusted nor adjusted models revealed a significant relationship. For categorical WWSDG duration, having any WWSDG (>0 hours) indicated a higher risk of hypertension, but confidence intervals were wide, reflecting uncertainty. Specifically, for WWSDG durations of 0-2 hours and ≥2 hours, there was a trend toward increased risk, but results remained non-significant.

**Table 5:** Associations between WWSDG and hypertension.

	Model 1 OR (95% CI)	Model 2 OR (95% CI)
WWSDG duration (continuous)	1.150 (0.982,1.351)	1.182 (0.894,1.546)
WWSDG (duration > 0 h)		
No	Reference	Reference
Yes	2.563 (0.827,7.944)	3.903 (0.947,16.090)
WWSDG duration (multi category)		
≤ 0 h	Reference	Reference
0–2 h	2.513 (0.748,8.449)	3.483 (0.940, 12.910)
≥ 2 h	2.632 (0.773,8.960)	5.089 (0.986, 23.382)

Model 1: no covariates were adjusted.

Model 2: weekday sleep duration, age, sex, race, income to poverty ratio and BMI were adjusted.

## 4 Discussion

This study aligns with recent evidence suggesting that the concept of “catch-up sleep” on weekends may not effectively mitigate the health risks associated with sleep deprivation accumulated during the week. Studies have questioned the physiological efficacy of weekend sleep extension as a means of “catching up” on lost sleep. For example, Putilov et al. demonstrated through sleep-wake cycle simulations that individuals do not significantly extend their sleep durations on weekends to compensate for weekday deficits (11)​. Furthermore, a recent study on university students found that health outcomes attributed to the WWSDG became statistically insignificant after controlling for weekday sleep duration, further underscoring the limited role of weekend sleep extension as a recovery mechanism (8)​.

Our findings reinforce the significance of sufficient weekday sleep for cardiovascular health in adolescents. Consistent with previous studies, we observed a higher hypertension risk in adolescents with weekday sleep durations below the recommended 8 hours. Insufficient sleep may contribute to hypertension through mechanisms such as increased sympathetic nervous activity, impaired glucose metabolism, and systemic inflammation. Unlike WWSDG, which merely reflects variability in sleep patterns, weekday sleep duration directly impacts the body’s physiological processes and may serve as a more reliable indicator of hypertension risk. These findings suggest that interventions should prioritize consistent sleep schedules and adequate weekday sleep over strategies focused solely on weekend sleep compensation.

Our research investigated the relationship between average weekday sleep duration, WWSDG, and the risk of hypertension in American teenagers. Our findings showed a direct link between short weekday sleep duration (less than 8 hours per night) and an increased risk of hypertension (OR = 1.092; 95% CI = 1.042-1.144). This aligns with recent epidemiological studies identifying a correlation between sleep patterns and hypertension. A study on Lithuanian adolescents aged 12 to 15 years found a significant relationship between insufficient sleep duration (less than 8 hours per day) and a higher prevalence of prehypertension and hypertension. Specifically, individuals who slept less than 7 hours daily had higher odds of high blood pressure (OR = 2.18 for prehypertension and 2.28 for hypertension) (12). In a study of 246 black and white adolescents (mean age = 15.7 years), wrist actigraphy over one week showed that shorter sleep durations were associated with higher 48-hour blood pressure, especially at night, resulting in a higher systolic blood pressure sleep/wake ratio. These associations were more pronounced among white adolescents (13). Among a cohort of 143 healthy-weight teenagers, a 7-day sleep diary indicated that inadequate sleep duration was associated with elevated systolic and diastolic blood pressure levels observed during various periods around nocturnal polysomnography (14). Polysomnography-determined sleep efficiency and total sleep time were inversely linked to blood pressure levels. However, occasional adequate sleep did not fully mitigate the risk of high blood pressure due to chronic sleep deprivation.

The mechanisms linking insufficient sleep and high blood pressure are not fully understood, but they involve factors such as the autonomic nervous system, inflammation, endothelial dysfunction, and circadian rhythm disruption. Sleep deprivation increases sympathetic activity and decreases parasympathetic activity, raising blood pressure. Research shows 24 hours of sleep deprivation raises blood pressure in young men, and similar deprivation increases systolic blood pressure in older adults, especially women (15). Sleep deprivation disrupts heart rate variability, impairing baroreflex function and raising blood pressure (16). Chronic sleep restriction activates inflammatory pathways, causing neuroinflammation and endothelial dysfunction (17, 18). Circadian rhythm disruption affects blood pressure regulation. Genes like Bmal1, Clock, Period, and Cryptochrome manage blood pressure, and their disturbances cause oxidative stress and inflammation, leading to hypertension (19). Research on shift workers and animals shows circadian disruption impacts blood pressure through these mechanisms.

Prior research has investigated the connection between WWSDG and hypertension. For instance, a study in South Korea found that an additional hour of sleep on weekends decreased the risk of developing hypertension by 17% across the general population and by 39% among individuals with insufficient sleep (7). Another study examined the impact of sleep duration, oversleeping, and weight on blood pressure in adolescents. An analysis of 327 children over two exam cycles showed that inadequate sleep, particularly on school nights, correlated with higher body weight and blood pressure, while oversleeping showed no such associations (20). Adolescents often experience a natural shift in their sleep patterns, staying up and waking up later due to biological and psychosocial influences such as social activities, academic demands, and technology use (21). However, early school start times and extracurricular commitments can disrupt these patterns, leading to sleep debt often compensated for by sleeping in on weekends. Discrepancies in sleep habits and living circumstances between youths and adults further complicate comparisons. Long-term studies focusing on children and adolescents are necessary to confirm the potential link between WWSDG and hypertension.

In analyzing WWSDG, we noted the presence of negative values in some cases, indicating shorter weekend sleep relative to weekdays. Such patterns may reflect individual lifestyle factors or circadian preferences rather than compensatory sleep behavior. For instance, adolescents with rigorous weekend schedules or social engagements may not extend their sleep on weekends, which results in a negative WWSDG. This variability highlights the importance of considering both lifestyle and circadian influences when interpreting WWSDG data.

This research is significant in its analysis of the correlation between weekend catch-up sleep and adolescent hypertension using data from NHANES. The data, derived from a substantial survey sample and weighted to reflect the general population, provide insights applicable to the broader demographic of American adolescents. Nonetheless, certain limitations must be acknowledged when interpreting the results. One primary constraint is the inability of our study to definitively establish a causal link between weekend catch-up sleep and hypertension due to its cross-sectional design. However, the findings do present opportunities for future longitudinal research to explore causal relationships. Additionally, the self-reported nature of adolescents’ sleep duration using a single unvalidated item is another limitation. While this method is commonly employed in population studies, there is a possibility of overestimation in self-reported sleep duration.

This study underscores the importance of promoting regular and sufficient sleep throughout the week for adolescent health. While adolescents may attempt to “catch up” on sleep during weekends, our findings suggest that such efforts may not mitigate the health risks associated with chronic weekday sleep loss. Public health campaigns and school policies should focus on promoting sleep hygiene practices that encourage adequate weekday sleep to reduce the risk of hypertension and other health complications in youth.

Future research should continue to explore the physiological impacts of sleep variability and assess whether other health outcomes are similarly unaffected by weekend sleep compensation. Longitudinal studies may provide further insights into how weekday sleep and WWSDG patterns develop over time and their long-term impact on cardiovascular health.

## 5 Conclusions

In conclusion, this study underscores the critical role of consistent and sufficient weekday sleep in mitigating the risk of hypertension among adolescents. The findings reveal that inadequate sleep during weekdays significantly heightens hypertension risk, while attempts to compensate for this deficit through weekend catch-up sleep provide no meaningful protective benefit. Longer total weekly sleep duration was associated with a reduced risk, highlighting the value of a stable sleep pattern throughout the week. Efforts to improve adolescent cardiovascular health should prioritize promoting regular and adequate sleep schedules, rather than relying on compensatory sleep strategies. Further longitudinal studies are warranted to explore causal relationships and assess the broader health implications of sleep variability.

## Data Availability

Publicly available datasets were analyzed in this study. This data can be found here: The survey data are publicly available on the internet for data users and researchers throughout the world (www.cdc.gov/nchs/nhanes/).
